# Essential Oils Extracted from Different Species of the Lamiaceae Plant Family as Prospective Bioagents against Several Detrimental Pests

**DOI:** 10.3390/molecules25071556

**Published:** 2020-03-28

**Authors:** Asgar Ebadollahi, Masumeh Ziaee, Franco Palla

**Affiliations:** 1 Moghan College of Agriculture and Natural Resources, University of Mohaghegh Ardabili, Ardabil 56199-36514, Iran; 2 Department of Plant Protection, Faculty of Agriculture, Shahid Chamran University of Ahvaz, Ahvaz 61357-43311, Iran; m.ziaee@scu.ac.ir; 3 Department of Biological, Chemical and Pharmaceutical Sciences and Technologies, University of Palermo, Palermo 38-90123, Italy

**Keywords:** essential oil, Lamiaceae, acute toxicity, sublethal effects, monoterpenoids

## Abstract

On the basis of the side effects of detrimental synthetic chemicals, introducing healthy, available, and effective bioagents for pest management is critical. Due to this circumstance, several studies have been conducted that evaluate the pesticidal potency of plant-derived essential oils. This review presents the pesticidal efficiency of essential oils isolated from different genera of the Lamiaceae family including *Agastache* Gronovius, *Hyptis* Jacquin, *Lavandula* L., *Lepechinia* Willdenow, *Mentha* L., *Melissa* L., *Ocimum* L., *Origanum* L., *Perilla* L., *Perovskia* Kar., *Phlomis* L., *Rosmarinus* L., *Salvia* L., *Satureja* L., *Teucrium* L., *Thymus* L., *Zataria* Boissier, and *Zhumeria* Rech. Along with acute toxicity, the sublethal effects were illustrated such as repellency, antifeedant activity, and adverse effects on the protein, lipid, and carbohydrate contents, and on the esterase and glutathione S-transferase enzymes. Chemical profiles of the introduced essential oils and the pesticidal effects of their main components have also been documented including terpenes (hydrocarbon monoterpene, monoterpenoid, hydrocarbon sesquiterpene, and sesquiterpenoid) and aliphatic phenylpropanoid. Consequently, the essential oils of the Lamiaceae plant family and their main components, especially monoterpenoid ones with several bioeffects and multiple modes of action against different groups of damaging insects and mites, are considered to be safe, available, and efficient alternatives to the harmful synthetic pesticides.

## 1. Introduction

Synthetic chemicals such as carbamates, pyrethroids, organochlorines, and organophosphates have played a principal role in the plant protection strategies against agroindustrial and medicinal arthropod pests within the past and present century. However, there are public concerns all over the world about the negative side effects of detrimental chemicals such as residues on food and in drinking water, acute or chronic toxicity to humans and other non-target organisms, outbreaks of secondary pests by abolishing their natural enemies, and the emergence of pest resistance [1,2,3]. Accordingly, there is an imperative demand to diminish chemical pesticides and reveal safe and potential alternatives.

In recent years, the utilization of biopesticides in pest management has been progressing. Three categories of biopesticides have been identified by the United State Environmental Protection Agency as follows: biochemical biopesticides such as botanical pesticides and other natural compounds, plant-incorporated protectants such as transgenic Bt toxin, and biocontrol organisms such as microbial fungi and parasitoid wasps [4]. Plants create secondary metabolites which include numerous aromatic compounds against different herbivores. They are considered to be attractants to pests’ antagonists and they can even be used by the plants to attract pollinators [5]. There are almost 17,500 aromatic plant species which are important for pesticidal approaches [6]. There is also ongoing progress in the search for pesticidal effects of essential oils extracted from different plant species for pest management strategies [7,8,9,10,11]. Furthermore, essential oils have been assayed as alternatives to commercial pesticides for the green conservation of cultural assets [12,13].

Although the susceptibility of many detrimental insects and mites to the essential oils extracted from different plant families has been documented, there are considerably many more reports on the high pesticidal potential of diverse species in the Lamiaceae family [14,15,16]. Therefore, the main objectives of this present study were to collect and review quality references in the field of pesticidal properties of essential oils extracted from the Lamiaceae plant family and to focus more on using natural agents to control harmful arthropods. This review was accumulated from journals indexed in major databases including Scopus, Web of Science, PubMed, and Google Scholar with separated and combined keywords of essential oil, Lamiaceae, toxicity, and lethal and sublethal effects. 

## 2. Plant Essential Oils and Their Pesticidal Activities

Plant essential oils, which are the principal reason for the odors of aromatic plants, are complex mixtures of terpenic (especially mono- and sesquiterpenes and mono- and sesquiterpenoids), aromatic, and aliphatic components with about 24 to 48 h half-lives, and are non-persistent in the environment [6,17]. The chemical composition of essential oils is affected by different endogenous and exogenous factors, for example, plant species, geographical position, climate conditions, harvesting time, and extraction method [18,19,20,21]. Plant essential oils or their volatile components cause critical defense strategies against herbivorous pests. They also have a vigorous role in plant–plant interactions and attraction of pollinators [22,23]. The environmental safety of essential oils is the main reason for their production in bulk quantities and their wide application in agriculture, cosmetic, and medical industries, and recently, in sustainable conservation of cultural assets [12,24,25]. Because of the natural origin of these secondary metabolites, they are degraded easily by natural degradation mechanisms [26]. Furthermore, apart from some exceptions, essential oils have not shown any toxicity to the homoeothermic animals and they are considered to be “generally recognized as safe” by the Environmental Protection Agency and Food and Drug Administration in the USA [27].

Essential oils exhibit a wide spectrum of pesticidal activities from lethal to sublethal effects against a wide range of insects and mites [7,24,28]. Pesticidal effects of essential oils extracted from different plant families such as Apiaceae, Asteraceae, Chenopodiaceae, Cupressaceae, Lamiaceae, Lauraceae, Myrtaceae, Zingiberaceae, Umbelliferae, and Geraniaceae have been documented [7,9,29,30]. Indeed, scientific attention to the application of essential oils for pest management strategies has witnessed progressive growth, in recent years [8]. Essential oils are biodegradable, commonly safe to mammals, and available agents [24]. Given that they are mixers of diverse components, various modes of action from acute toxicity to repellency, antinutritional and developmental inhibitory effects, and the impact on neural and biochemical processes have been recorded for them [16,26,31]. Therefore, there is slight change of pests’ resistance to these botanical volatiles [32], and it can be concluded that these plant-derived volatiles are eco-friendly and effective alternatives to the detrimental synthetic pesticides.

## 3. Lamiaceae Plant Family with Potential Pesticidal Oils 

The Lamiaceae or mint family with a wide distribution in different natural ecosystems comprises 236 genera and about 6000 species [33]. These aromatic plants have square stems in cross-section, opposite leaves, zygomorphic flowers with five united petals and sepals, and they are cultivated because of their easy propagation, i.e., stem cutting [34]. Many species from the Lamiaceae family possess high quality essential oils in all aboveground parts, especially in leaves and flowers, and are distinguished in applications for medicinal purposes with effective antibacterial, antifungal, antioxidant, antiviral, and even anticancer properties, as well as in the cosmetic and perfumery industries [35,36,37].

The search in large scientific databases including Scopus, Web of Science, PubMed, and Google Scholar displayed that Plant genera of the family Lamiaceae including *Agastache*, *Hyptis*, *Lavandula*, *Lepechinia*, *Mentha*, *Melissa*, *Ocimum*, *Origanum*, *Perilla*, *Perovskia*, *Phlomis*, *Rosmarinus*, *Salvia*, *Satureja*, *Teucrium*, *Thymus*, *Zataria*, and *Zhumeria* have great potential in pest management strategies. According to Table 1, pesticidal effects of the essential oils isolated from these plants can be expressed by lethal (acute toxicity) to sublethal (chronic effects) approaches including repellent and antifeedant activities, and adverse effects on the energy content and digestive enzymes or the enzymes that are effective in electron transport. According to the many reports on the insecticidal and acaricidal activities, *Lavandula*, *Mentha*, *Ocimum*, *Origanum*, *Satureja*, and *Thymus* species are among the most promising plants in detrimental pest management [38,39,40,41,42,43,44,45,46,47].

## 4. Chemical Composition of Essential Oils and Their Relative Pesticidal Effects

On the basis of the total percentage, the main two to four components of the introduced Lamiaceae essential oils are displayed in Table 2. Some of these components such as 1,8-cineole, borneol, camphor, carvacrol, caryophyllene, linalool, terpinene, thymol, α-pinene, β-pinene, and ρ-cymene were identified in most of the species. For example, 1,8-cineole was introduced as one of the main components of *Agastache foeniculum* (Pursh) Kuntze, *Lavandula angustifolia* Miller, *Lavandula hybrida* Briq., *Lavandula luisieri* (Rozeira) Rozeira, *Mentha microphylla* C. Kock, *Mentha spicata* L., *Perovskia atriplicifolia* Benth., *Rosmarinus officinalis* L., *Salvia apiana* Jepson, *Salvia multicaulis* Vahl., *Salvia pratensis* L., and *Satureja hortensis* L. essential oils. In contrast, some others were found as main components in the unique species. Sclareol, for instance, was reported as a major component only in the *Salvia sclarea* L. essential oil (Table 2).

**Figure 1:**
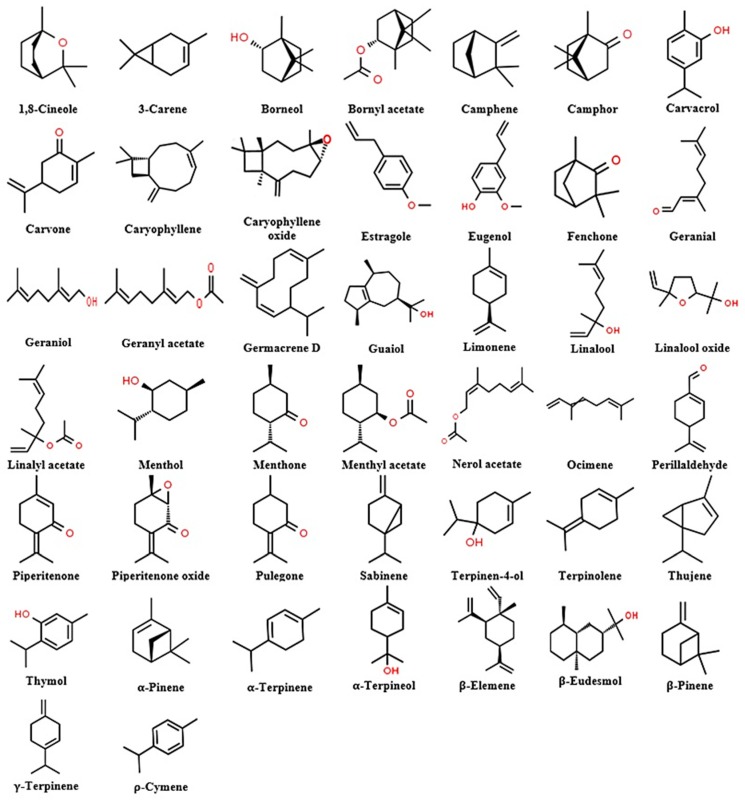
Chemical structure of main components identified in the essential oils of the Lamiaceae plant family.

Terpenes which are large and diverse natural hydrocarbons are commonly classified according to the number of isoprene units C_5_H_8_, to hemi- (one unit), mono- (two units), sesqui- (three units), di- (four units), ses- (five units), tri- (six units), tetra- (8 units), and polyterpenes (n units) [85]. Although the majority of these compounds are found in plant essential oils, the more complex terpenes, such as lanosterol, exist in animals. Furthermore, each terpene can be oxygenated and modified to terpenoid hydrocarbons [86,87]. In general, there are high amount of terpenes in the essential oils of the Lamiaceae family. The main components in the essential oils of the Lamiaceae plants from acyclic, cyclic, and bicyclic monoterpenes; monoterpenoids; sesquiterpenes; sesquiterpenoids; and phenylpropanes’ groups [88] possess promising pesticidal properties against several arthropod pests (Table 3 and Figure 1).

Table 3 summarizes the bioactivity of the main components identified in the essential oils of Lamiaceae as a pesticide agent against insect pests. It has been found that the monoterpenoids had more fumigant toxicity than monoterpenes against the maize weevil, *Sitophilus zeamais* Motschulsky (Coleoptera: Curculionidae) [90]. The ketone, aldehyde (such as camphor, carvone, fenchone, and menthone), and epoxide (such as 1,8-cineole and limonene oxide) derivatives of monoterpenoids have also been found to be more toxic than alcohol and ester groups (such as borneol, linalool, and menthol) [90]. The essential oil of *Salvia leriifolia* Benth (Lamiaceae) showed the highest insecticidal activity as fumigant and contact agents against adults of *C. maculatus, S. oryzae,* and *T. castaneum* [100]. It was indicated that among eight terpenes including 1,8-cineole, carvacrol, eugenol, menthone, linalool, limonene, β-pinene, and α-pinene, the cyclic monoterpenoids carvacrol and menthone had the most fumigant toxicity against the cowpea weevil, *C. maculatus* [101]. In another study, the fumigant and contact toxicities of eugenol (a phenylpropanoid), ρ-cymene and α-pinene (monoterpenes), and menthone, α-terpinene, and terpinen-4-ol (monoterpenoids) were evaluated against *T. castaneum* adults. Terpinen-4-ol and α-terpinene as cyclic terpenes had the most fumigant toxicity followed by menthone, ρ-cymene, α-pinene, and eugenol [96]. In contrast, eugenol was the most toxic compound in the contact bioassay. The fumigant toxicity of some essential oils’ components was evaluated against bed bugs (*Cimex lectularius* L.) and it was found that cyclic monoterpenoids thymol and carvacrol had much more fumigant toxicity than bicyclic monoterpenoids camphor and 1,8-cineole, cyclic monoterpene limonene, bicyclic monoterpene α-pinene, and phenylpropanes cinnamaldehdye, citronellic acid, eugenol and methyl eugenol [93]. Consequently, although the high insecticidal effects of monoterpenes, monoterpenoids, sesquiterpenes, sesquiterpenoids, and phenylpropanes has been reported, on the one hand, the monoterpenoids, especially cyclic monoterpenoids, showed the most toxic effects. On the other hand, minor structural differences cause major alterations in the toxic effects. However, susceptibility of the considered pests can be affected by the synergistic effects of other minor components [102].

Apart from insecticidal activity, the acaricidal efficacy of essential oils extracted from some Lamiaceae species have also been reported. The acaricidal effects of the essential oils of *S. hortensis, Mentha pulegium* L., *Mentha viridis* L., *R. officinalis*, and *Z. multiflora* was demonstrated against *Tetranychus turkestani* Ugarov and Nikolskii (Acari: Tetranychidae) [41]. Some of the constituents of *R. officinalis* essential oil were toxic against *Tetranychus urticae* Koch. (Acari: Tetranychidae) on bean and tomato plants. However, a synergistic effect among the active and inactive constituents was observed when they were mixed with each other [103]. 

Since larva is the damaging stage of insects, evaluating the larvicidal effect of essential oils is of great importance. For example, the larvicidal potential of essential oils of different Lamiaceae species has been reported in literature studies. The oil of *Mentha piperita* L. (Lamiaceae) was found to be an effective larvicidal agent against the housefly, *Musca domestica* (L.) (Diptera: Muscidae) [104]. The essential oil of *M. piperita* had higher larvicidal pupicidal than *M. citrata* Ehrh oil in contact and fumigant applications against *M. domestica* [105]. Similar results were obtained by the essential oil of *M. piperita* which had the most promising larvicidal against *Anopheles stephensi* Liston and *Aedes aegypti* L. (Diptera: Anophelinae) among 25 tested plant essential oils [106]. The larvicidal effects of *M. piperita* essential oil with a high quantity of bioactive monoterpenes were also reported on the *M. domestica* and *An. stephensi* larvae [107]. In other research with pure components, larvicidal effects of fifty constituents from terpene, terpenoid, and phenylpropanoid groups against *Culex quinquefasciatus* Say were assessed and it was demonstrated that carvacrol and thymol as cyclic monoterpenoids were the most toxic against larvae among all tested components [89].

Most of the Lamiaceae essential oils also have high repellent activity toward insects. For example, the essential oils of *M. piperita* and *M. citrata* showed effective repellency against *M. domestica* populations [104]. The repellent effect of essential oils extracted from *Salvia dorisiana* Standl, *S. longifolia* Nutt, and *S. sclarea* were reported against *Aedes albopictus* (Diptera:Culicidae) [108]. *Ephestia kuehniella* (Zeller) (Lepidoptera: Pyralidae) and *Plodia interpunctella* (Hübner) (Lepidoptera: Pyralidae) were also significantly repelled by *Thymus daenensis* Celak essential oil [109]. The essential oil of *M. pulegium* caused high repellent activity against the adults of *T. castaneum* and *Lasioderma serricorne* (F.) (Coleoptera: Anobiidae) [110]. Furthermore, substantial repellency of *M. piperita* essential oil on *M. domestica* has been reported which was higher than *Eucalyptus globulus* Labill and *Citrus sinensis* (L.) Osbeck essential oils [111].

## 5. Mode of Pesticidal Action

The mechanism of action of Lamiaceae essential oils and their components on pests is not completely recognized but, based on their diverse lethal and sublethal effects, it is obvious that these natural agents affect in different ways. The difference in the pesticidal potential of these agents could also be related to differences in their structures. For example, the higher toxicity of oxygenated monoterpenes as compared with the non-oxygenated ones could be due to the different structures of these components. Furthermore, according to recent findings, different physiological and behavioral modes of action of essential oils and their components have been reported. For example, inhibition of adenosine triphosphatases (ATPases), acetylcholinesterase (AChE) and butyrylcholinesterase (BuChE) [76,96], and distribution in the octopamine and gamma-aminobutyric acid receptors (GABArs) have been documented [112]. Given that octopamine receptors are invertebrates-specific, the use of essential oils in pest management, with this mode of action, can be considered to be safe bioagents for mammals [112]. Some other studies revealed that they can diminish esterase and glutathione S-transferases (GSTs) activities and total carbohydrate, lipid, and protein contents in the pests [73,113]. Detoxifying enzymes such as esterase and glutathione S-transferases have a significant effect on pest resistance before pesticides. Consequently, impairing the function of such enzymes can reduce the likelihood of pest resistance to essential oils [114]. Furthermore, histological changes in the epithelial cells of insects’ midguts [115], and even a diminution in the respiration rate of pests have also been reported [116]. Generally, because of the wide pesticidal effects and several modes of action of the essential oils and their derivatives, pests have very few changes to resistant before these safe agents, and therefore they are very valuable in pest management strategies. 

## 6. Conclusions

The overuse of synthetic chemicals in pest management programs has caused several side effects, such as contamination of drinking water; residues on food; acute or chronic negative effects on mammals and non-target organism including bird, bees, parasitoids, and predators; and the development of pest resistance. Due to this circumstance, researchers have focused on the application of plant-derived essential oils from different plant genera and families in recent years. Although the pesticidal properties of essential oils extracted from different species of Apiaceae, Lamiaceae, Myrtaceae, Rutaceae, Verbenaceae, and Zingiberaceae families have been recognized, the present review focused on the Lamiaceae species based on their availability and diversity. Along with these advantages, plant essential oils generally are nontoxic to mammals and other vertebrates. Furthermore, based on their multiple modes of action, development of pests’ resistance against them is very low. Consequently, the Lamiaceae plant essential oils and their components with a wide range of lethal and sublethal effects against different damaging insects and mites in the field, greenhouse and storage conditions, have great potential in pests’ management strategies and are considered to be safe, available, and eco-friendly alternatives to the synthetic chemicals. Indeed, the most important issue to be considered for the application of essential oils is their rapid degradation under the influence of air and light, which could be overcome by encapsulation or controlled release techniques. Briefly, the essential oils and their components, as core materials, are protected from adverse environmental factors in these techniques. Emulsifying the essential oils and components through adjuvants is another solution to improve pesticidal efficiency. However, their direct and indirect effects on the other non-target organisms such as honeybees and natural biocontrol agents and the economic aspects must be assessed before commercialization.

## Figures and Tables

**Table 1:** A list of reports indicating pesticidal effects of essential oils isolated from the Lamiaceae family.

Genera	Species	Lethal/SubLethal Effects and Targeted Arthropod Pests
*Agastache* Gronovius	*A. foeniculum* (Pursh) Kuntze	Fumigant toxicity against *T. castaneum* and *R. dominica* [42].
*Hyptis* Jacquin	*H. spicigera* Lamarck	Repellency against *S. zeamais* [43].
	*H. suaveolens* (L.) Poit	Repellency against *S. zeamais* [43].
*Lavandula* L.	*L. angustifolia* Miller	Contact and fumigant toxicity against *T. urticae* [44].
	*L. hybrida* Reverchon	Repellency against *S. zeamais*, *C. pusillus*, and *Tenebrio molitor* [42].
	*L. luisieri* (Rozeira) Rozeira	Antifeedant effects against *L. decemlineata* [43].
	*L. stoechas* L.	Fumigant toxicity against *T. castaneum*, *L. serricorne*, and *R. dominica* [47].
*Lepechinia* Willdenow	*L. betonicifolia* (Lam.) Epling	Repellency against *T. castaneum* [48].
Mentha L.	*M. arvensis* L.	Acaricidal effects on *D. farinae* and *D. pteronyssinus* [49].
	*M. longifolia* (L.) L.	Larvicidal activity against *C. pipiens* [50].
	*M. microphylla* Koch	Contact and fumigant toxicity against *T. castaneum* and *S. oryzae* [51].
	*M. piprita* L.	Toxicity on the adult females of *A. gossypii* [52].
	*M. pulegium* L.	Toxicity on the adult females of *A. gossypii* [52].
	*M. spicata* L.	Larvicidal activity against *C. pipiens* [50].
	*M. suaveolens* Ehrh.	Larvicidal activity against *C. pipiens* [50].
Melissa L.	*M. officinalis* L.	Fumigant toxicity and antifeedant effect against *T. castaneum* [53].
*Ocimum* L.	*O. americanum* L.	Toxicity and repellency against *S. zeamais* [54].
	*O. basilicum* L.	Contact and fumigant toxicity against *T. putrescentiae* [55].
	*O. gratissimum* L.	Larvicidal activity against *C. quinquefasciatus* [56].
	*O. sanctum* L.	Larvicidal activity against *C. quinquefasciatus* [56].
*Origanum* L.	*O. acutidens* Ietswaart	Toxicity effects on adults of *Bruchus dentipes* [57].
	*O. elongatum* E. & M.	Acaricidal activity against *V. destructor* [58].
	*O. glandulosum* Desf.	Contact and fumigant toxicities against *R. dominica* [59].
	*O. onites L.*	Contact and fumigant toxicity against *R. dominica*, *T. confusum*, *S. granarius*, and *S. oryzae* [60].
	*O. rotundifolium* Boiss.	Fumigant toxicity against *S. granaries* adults [61].
	*O. vulgare* L.	Exposure to volatile compounds against *A. punctatum* [13].
*Perilla* L.	*P. frutescens* (L.) Britton	Insecticidal and repellent activities against *L. serricorne* [62].
*Perovskia* Kar.	*P. atriplicifolia* Benth.	Fumigant toxicity against *S. oryzae* and *T. castaneum* adults [63].
*Phlomis* L.	*P. umbrosa* Turcz.	Contact and fumigant toxicity on *S. zeamais* and *T. castaneum* [64].
*Rosmarinus* L.	*R. officinalis* L.	Contact and fumigant toxicity, and repellency effects against *S. longipalpa* [65].
*Salvia* L.	*S. fruticosa* Mill.	Larvicidal activity against *C. pipiens* [50].
	*S. hydrangea* Dc.	Fumigant toxicity against *S. granaries* adults [61].
	*Salvia apiana* Jeps.	Deterrent and larvicidal activity on *A. aegypti* and *A. quadrimaculatus* [66].
	*Salvia elegans* Vahl	Deterrent and larvicidal activity on *A. aegypti* and *A. quadrimaculatus* [66].
	*Salvia leucantha* Cav.	Deterrent and larvicidal activity on *A. aegypti* and *A. quadrimaculatus* [66].
	*S. multicaulis* Vahl.	Fumigant toxicity against adults of *S. granaries* [61].
	*S. numerosa* L.	Fumigant toxicity against adults of *S. granaries* [61].
	*S. officialis* L.	Deterrent and larvicidal activity on *A. aegypti* and *A. quadrimaculatus* [66].
	*S. pomifera* Hayek	Larvicidal activity against *C. pipiens* [50].
	*S. pratensis* L.	Contact and fumigant toxicity against *T. castaneum* and *C. maculatus* [67].
	*S. sclarea* L.	Fumigant toxicity against adults of *S. granaries* [61].
*Satureja* L.	*S. hortensis* L.	Contact and fumigant toxicity against *T. urticae* [68].
	*S. khuzistanica* Jamzad	Antifeedant activity and toxicity to *L. decemlineata* [69].
	*S. spicigera* Boiss.	Fumigant toxicity against adults of *S. granaries* [61].
	*S. thymbra* L.	Fumigant toxicity against *E. kuehniella, P. interpunctella* and *A. obtectus* [70].
*Teucrium* L.	*T. polium* L.	Contact and fumigant toxicity against *T. urticae* [68].
*Thymus* L.	*T. daenensis* Celak	Antifeedant activity and toxicity to *L. decemlineata* [69].
	*T. eriocalyx* (Ronniger) Jalas	Contact and fumigant toxicity against *T. urticae* [71].
	*T. fallax* Fisch. & Mey.	Fumigant toxicity against adults of *S. granaries* [61].
	*T. kotschyanus* Boiss.	Contact and fumigant toxicity against *T. urticae* [71].
	*T. persicus* (Ronniger ex Rech.f.) Jalas	Fumigant toxicity against adults of *T. castaneum* and *S. oryzae* [69].
	*T. satureioides* C. & B.	Acaricidal activity against *V. destructor* [58].
	*T. sipyleus* Boiss.	Fumigant toxicity against adults of *S. granaries* [61].
	*T. vulgaris* L.	Exposure to volatile compounds against *A. punctatum* [42].
*Zataria* Boissier	*Z. multiflora* Boiss.	Fumigant toxicity on adults of *T. castaneum*, *S. granarius* and *C. maculatus* [72].
*Zhumeria* Rech.	*Z. majdae* Rech.	Adverse effect on protein, lipid and carbohydrate contents and on esterase and glutathione S-transferase enzymes’ activities of *T. castaneum* larvae [73].

The full scientific names of mentioned pests are as follows: *Acanthoscelides obtectus* Say (Coleoptera: Chrysomelidae), *Aedes aegypti* L. (Diptera: Culicidae), *Anobium punctatum* de Geer (Coleoptera: Anobidae), *Anopheles dirus* Peyton & Harrison (Diptera: Culicidae), *Anopheles quadrimaculatus* Say (Diptera: Culicidae), *Aphis gossypii* Glover (Hemiptera: Aphididae), *Brevicoryne brassicae* L. (Hemiptera: Aphididae), *Bruchus dentipes* Baudi (Coleoptera: Chrysomelidae), *Callosobruchus maculatus* (F.) (Coleoptera: Chrysomelidae), *Culex quinquefasciatus* Say. (Diptera: Culicidae), *Culex pipiens* L. (Diptera: Culicidae), *Cryptolestes pusillus* (Schöenherr) (Coleoptera: Laemophloeidae), *Dermatophagoides farinae* Hughes (Acari: Pyroglyphidae), *Dermatophagoides pteronyssinus* (Trouessart) (Acari: Pyroglyphidae), *Ephestia kuehniella* Zeller (Lepidoptera: Pyralidae), *Lasioderma serricorne* F. (Coleoptera: Anobiidae), *Leptinotarsa decemlineata* (Say) (Coleoptera: Chrysomelidae), *Musca domestica* L. (Diptera: Muscidae), *Plodia interpunctella* Hübner (Lepidoptera: Pyralidae), *Rhyzopertha dominica* (F.) (Coleoptera: Bostrichidae), *Sitophilus granarius* (L.) (Coleoptera: Curculionidae), *Sitophilus oryzae* (L.) (Coleoptera: Curculionidae), *Sitophilus zeamais* Motschulsky (Coleoptera: Curculionidae), *Supella longipalpa* (Fabricius) (Blattaria: Ectobiidae), *Tenebrio molitor* L. (Coleoptera: Tenebrionidae), *Tetranychus urticae* Koch (Acari: Tetranychidae), *Tribolium castaneum* Herbst (Coleoptera: Tenebrionidae), *Tyrophagus putrescentiae* (Schrank) (Sarcoptiformes: Acaridae) and *Varroa destructor* Anderson & Trueman (Acari: Varroidae).

**Table 2:** Main components of the essential oils isolated from different species of the Lamiaceae plant family with pesticidal prominence.

Species	Main Components (percentage)
*Agastache foeniculum*	Estragole (94.0), 1,8-cineole (3.3), 1-octen-3-ol (0.5), and germacrene D (0.4) [42].
*Hyptis spicigera*	α-Pinene (21.7), caryophyllene (18.4), sabinene (17.4), and β-pinene (13.8) [43].
*Hyptis suaveolens*	Sabinene (27.0), caryophyllene (17.1), terpinolene (11.9), and β-pinene (9.4) [43].
*Lavandula angustifolia*	Linalool (28.6), 1,8-cineole (18.6), borneol (15.9), and camphor (8.2) [44].
*Lavandula hybrid*	Linalool (37.3), linalyl acetate (24.6), 1,8-cineole (9.9), and camphor (6.5) [45].
*Lavandula luisieri*	1,8-Cineole (26.3), nerol acetate (17.5), α-necrodo (8.2), and fenchone (6.6) [74].
*Lavandula stoechas*	Fenchone (41.9), camphor (34.6), α-pinene (2.8), and linalool (2.7) [74].
*Lepechinia betonicifolia*	Limonene (27.5), α-pinene (19.4), β-pinene (9.5), and caryophyllene (6.8) [48].
*Mentha arvensis*	Menthol (59.8), menthone (20.0), menthyl acetate (6.5), and pulegone (2.8) [49].
*Mentha longifolia*	Carvone (54.7), limonene (20.0), β-pinene (5.0), and piperitenone (5.0) [50].
*Mentha microphylla*	Piperitenone oxide (46.7), piperitone oxide (28.0), and 1,8-cineole (13.3) [51].
*Mentha piprita*	Limonene (27.3), menthol (24.7), menthone (14.0), and carvone (8.5) [52].
*Mentha pulegium*	Pulegone (73.4), piperitenone (5.5), decane (5.0), and limonene (3.1) [52].
*Mentha spicata*	Piperitenone oxide (35.7), 1,8-cineole (14.5), calamene (6.4), and viridiflorol (4.3) [50].
*Mentha suaveolens*	Piperitenone oxide (62.7), α-pinene (3.4), limonene (3.3), and ρ-cymene (2.6) [50].
*Melissa officinalis*	γ-Terpinene (47.9), carvacrol (31.4), α-terpinene (5.2), and ρ-cymene (4.3) [53].
*Ocimum americanum*	Methyl eugenol (53.9), eugenol (23.9), caryophyllene (17.7), and β-chamigene (2.2) [54].
*Ocimum basilicum*	Estragole (86.3), α-bergamotene (5.9), ocimene (2.5), and β-elemene (1.9) [54].
*Ocimum gratissimum*	Methyl eugenol (64.4), ocimene (10.4), and caryophyllene (5.1) [75].
*Ocimum sanctum*	α-Cubebene (12.5), geranial (12.3), caryophyllene (10.8), and α-bisabolene (10.2) [54].
*Origanum acutidens*	Carvacrol (86.9), γ-terpinene (0.7), and ρ-cymene (1.9) [57].
*Origanum elongatum*	Carvacrol (67.3), γ-terpinene (9.3), thymol (9.2), and ρ-cymene (4.2) [58].
*Origanum glandulosum*	Thymol (38.8), carvacrol (32.9), ρ-cymene (7.9), and γ-terpinene (5.1) [59].
*Origanum onites*	Thymol (22.9), γ-terpinene (13.0), ρ-cymene (12.9) and carvacrol (7.2) [60].
*Origanum rotundifolium*	Carvacrol (56.8), ρ-cymene (13.1), ocimene (5.4), and caryophyllene (3.9) [76].
*Origanum vulgare*	Thymol (27.18), *p*-cymene (18.97), and carvacrol (4.04) [13].
*Perilla frutescens*	Carvone (32.6), perilla aldehyde (20.5), and caryophyllene (9.9) [62].
*Perovskia atriplicifolia*	Camphor (28.4), 1,8-cineole (23.2), 3-caren (7.5), and α-pinene (6.7) [63].
*Phlomis umbrosa*	Geranial (16.5), linalool (13.3), geraniol (7.4), and caryophyllene (6.3) [64].
*Rosmarinus officinalis*	α-Pinene (19.6), 1,8-cineole (9.1), limonene (8.2), and camphene (3.8) [48].
*Salvia apiana*	1,8-Cineole (71.7), α-pinene (5.1), camphor (4.4), and β-pinene (3.8) [66].
*Salvia elegans*	Borneol (17.4), β-eudesmol (10.4), bornyl acetate (5.0), and guaiol (4.8) [66].
*Salvia fruticosa*	Camphor (23.1), α-pinene (12.7), borneol (12.6), and camphene (9.0) [50].
*Salvia hydrangea*	caryophyllene(33.4) and caryophyllene oxide (25.4) [77].
*Salvia leucantha*	Bornyl acetate (11.4), caryophyllene (6.5), caryophyllene oxide (13.5), and spathulenol (7.0) [66].
*Salvia multicaulis*	1,8-Cineole (17.0), α-pinene (11.5), caryophyllene (8.9), and ρ-cymene (3.7) [78].
*Salvia numerosa*	Sabinene (37.0), germacrene D (9.0), caryophyllene (8.0), and caryophyllene oxide (2.6) [79].
*Salvia officialis*	α-Thujene (25.8), viridiflorol (20.4), β-thujene (5.7), and camphor (6.4) [66].
*Salvia pomifera*	Terpinen-4-ol (15.8), caryophyllene oxide (13.2), sabinene (12.9), and β-pinene (12.1) [50].
*Salvia pratensis*	Dodecane (30.4), tridecane (12.1), Undecane (11.9), and 1,8-cineole (6.3) [67].
*Salvia sclarea*	Sclareol (11.0), germacrene D (9.8), caryophyllene (9.0), and α-terpineol (7.4) [78].
*Satureja hortensis*	Oleic acid (17.0), thymol (16.5), palmitic acid (12.7), and 1,8-cineole (10.9) [68].
*Satureja khuzistanica*	Carvacrol (81.1), ρ-cymene (3.3), β-bisabolene (2.7), and γ-terpinene (2.3) [69].
*Satureja spicigera*	Carvacrol (53.7), thymol (36.0), and caryophyene oxide (6.1) [80].
*Satureja thymbra*	Carvacrol (53.7), γ-terpinene (17.6), thymol (13), and ρ-cymene (10.1) [70].
*Teucrium polium*	Lycopersene (26.0), dodecane (17.5), tridecane (7.4), and undecane (7.2) [68].
*Thymus daenensis*	Thymol (72.3), carvacrol (7.1), ρ-cymene (5.4), and γ-terpinene (4.8) [69].
*Thymus eriocalyx*	Thymol (28.8), oleic acid (11.5), palmitic acid (8.6), and borneol (5.7) [71].
*Thymus fallax*	Carvacrol (66.1), ρ-cymene (7.1), ocimene (5.5), and γ-terpinene (4.6) [81].
*Thymus kotschyanus*	Camphene (35.6), linalyl acetate (20.5), linalool (14.8), and α-terpineol (13.9) [71].
*Thymus persicus*	Carvacrol (44.7) thymol (11.0), terpinen-4-ol (8.12), and α-pinene (6.2) [82].
*Thymus satureioides*	Borneol (36.6), α-terpineol (15.8), camphene (8.9), and α-pinene (4.3) [58].
*Thymus sipyleus*	Thymol (38.3), carvacrol (38.0), γ-terpinene (7.28%) and ρ-cymene (4.2) [83].
*Thymus vulgaris*	Carvacrol (64.96), thymol (8.25), and *p*-cymene (11.29) [13].
*Zataria multiflora*	Thymol (47.5), ρ-cymene (13.2), carvacrol (9.6), and linalool (7.9) [84].
*Zhumeria majdae*	Linalool (58.3), camphor (25.9), linalool oxide (1.5), and borneol (1.1) [73].

**Table 3:** Classification and pesticidal effects of main components reported in the essential oils of the Lamiaceae plant family.

Components	Classification	Pesticidal Effects
1,8-Cineole	Bicyclic monoterpenoid	Larvicidal and pupicidal activity against *C. quinquefasciatus* [89].
3-Carene	Bicyclic monoterpene	Toxicity against the adults of *S. zeamais* [90].
Borneol	Bicyclic monoterpenoid	Larvicidal and pupicidal activity against *C. quinquefasciatus* [89].
Bornyl acetate	Bicyclic monoterpenoid	Fumigant toxicity against the adults of *S. granaries* [91].
Camphene	Bicyclic monoterpene	Toxicity on *P. xylostella* larvae [92].
Camphor	Bicyclic monoterpenoid	Larvicidal and pupicidal activity against *C. quinquefasciatus* [89].
Carvacrol	Cyclic monoterpenoid	Contact and fumigant toxicity against the adults of *C. lectularius* [93].
Carvone	Cyclic monoterpenoid	Toxicity against the adults of *S. zeamais* [90].
Caryophyllene	Bicyclic sesquiterpene	Insecticidal activities against *S. frugiperda* larvae and pupae [94].
Caryophyllene oxide	Bicyclic sesquiterpenoid	Insecticidal activities against *S. frugiperda* larvae and pupae [94].
Estragole	Cyclic phenylpropanoid	Fumigant toxicity and acetylcholine esterase inhibition against *B. germanica* [95].
Eugenol	Cyclic phenylpropanoid	Contact toxicity against the adults of *T. castaneum* [96].
Fenchone	Bicyclic monoterpenoid	Fumigant toxicity against the adults of *S. granaries* [91].
Geranial	Acyclic monoterpenoid	Larvicidal and pupicidal activity against *C. quinquefasciatus* [89].
Geraniol	Acyclic monoterpenoid	Contact and fumigant toxicity against the adults of *C. lectularius* [93].
Geranyl acetate	Acyclic monoterpenoid	Fumigant toxicity against the adults of *S. granaries* [91].
Germacrene D	Cyclic sesquiterpene	Larvicidal and pupicidal activity against *C. quinquefasciatus* [89].
Guaiol	Bicyclic sesquiterpenoid	Fumigant toxicity against the adults of *S. granaries* [91].
Limonene	Cyclic monoterpene	Toxicity against the adults of *S. zeamais* [90].
Linalool	Acyclic monoterpenoid	Toxicity on *P. xylostella* larvae [92].
Linalool oxide	Cyclic monoterpenoid	Toxicity on *P. xylostella* larvae [92].
Linalyl acetate	Acyclic monoterpenoid	Fumigant toxicity against the adults of *S. granaries* [91].
Menthol	Cyclic monoterpenoid	Fumigant toxicity against the adults of *S. granaries* [91].
Menthone	Cyclic monoterpenoid	Contact and fumigant toxicity against the adults of *C. lectularius* [93].
Menthyl acetate	Cyclic monoterpenoid	Fumigant toxicity and repellent activity on first-instar nymphs of *R. prolixus* [97].
Nerol acetate	Acyclic monoterpenoid	Fumigant toxicity against the adults of *S. granaries* [91].
Ocimene	Acyclic monoterpene	Fumigant toxicity and acetylcholine esterase inhibition against *B. germanica* [95].
Perillaldehyde	Cyclic monoterpenoid	Larvicidal and pupicidal activity against *C. quinquefasciatus* [89].
Piperitenone	Cyclic monoterpenoid	Larvicidal and pupicidal activity against *C. quinquefasciatus* [89].
Piperitenone oxide	Cyclic monoterpenoid	Larvicidal, ovicidal, oviposition-deterrent, and repellent effect against *A. stephensi* [98].
Pulegone	Cyclic monoterpenoid	Larvicidal and pupicidal activity against *C. quinquefasciatus* [89].
Sabinene	Bicyclic monoterpene	Larvicidal and pupicidal activity against *C. quinquefasciatus* [89].
Terpinen-4-ol	Cyclic monoterpenoid	Contact and fumigant toxicity against *C. lectularius* adults [93].
Terpinolene	Cyclic monoterpene	Larvicidal and pupicidal activity against *C. quinquefasciatus* [89].
Thujene	Bicyclic monoterpene	Fumigant toxicity and acetylcholine esterase inhibition against *B. germanica* [89].
Thymol	Cyclic monoterpenoid	Fumigant toxicity against the adults of *S. granaries* [91].
α-Pinene	Bicyclic monoterpene	Fumigant and contact toxicities and repellency against *T. castaneum* adults [96].
α-Terpinene	Cyclic monoterpene	Larvicidal and pupicidal activity against *C. quinquefasciatus* [89].
α-Terpineol	Cyclic monoterpenoid	Fumigant toxicity against the adults of *S. granaries* [91].
β-Elemene	Cyclic sesquiterpene	Contact toxicity against *D. melanogaster* [99].
β-Eudesmol	Bicyclic sesquiterpenoid	Contact toxicity against *D. melanogaster* [99].
β-Pinene	Bicyclic monoterpene	Fumigant toxicity against the adults of *S. granaries* [91].
γ-Terpinene	Cyclic monoterpene	Larvicidal and pupicidal activity against *C. quinquefasciatus* [89].
ρ-Cymene	Cyclic monoterpene	Larvicidal and pupicidal activity against *C. quinquefasciatus* [89].
